# Effect of physical activity on social communication impairments in children with autism spectrum disorder: A meta-analysis

**DOI:** 10.1016/j.heliyon.2024.e39053

**Published:** 2024-10-09

**Authors:** Kai Qi, Xiaoshuang Wang, Qi Xu, Bingyu Hu, Ziyi Wang, Marcin Białas

**Affiliations:** a Gdansk University of Physical Education and Sport, 80-336, Gdańsk, Poland; b College of Physical Education, Chizhou University, Chizhou, 247000, Anhui, China; c Yanshan University, Qinhuangdao, 066000, Hebei, China

**Keywords:** Autism spectrum disorder, Social communication impairments, Children, Physical activity

## Abstract

This meta-analysis aims to systematically evaluate if different physical activities (PA) improve the social communication impairments (SCI) seen in children with autism spectrum disorder (ASD). For this meta-analysis, five databases (Web of Science, PubMed and Cochrane Library database in English, and CNKI and Wanfang Data Knowledge Service Platform in Chinese) were searched from database inception to September 11, 2024. The eligibility criteria included (1) study population comprised children with ASD, with no gender restriction; (2) experimental group consisted of a PA intervention; (3) control group consisted of nonPA interventions or routine activities; (4) outcomes were assessed using validated SCI scales (e.g., social cognition, social motivation, and/or social awareness); and (5) study design involved a randomized parallel group design. The quality of the evidence for each study was assessed using the Cochrane Risk of Bias tool. From a pool of 2714 potentially relevant articles, 17 were eligible for inclusion in this review. The results of overall response of PA intervention to SCI illustrated a Medium degree of statistical heterogeneity between studies (I^2^ = 53.3 %, p < 0.01); the effect size of PA intervention on SCI (expressed as standardized mean difference) was −0.34 (95 % CI: −0.57, −0.11), which was statistically significant. SCI of autistic children could be potentially improved by PA interventions, though further research is needed to clarify if benefits achieve clinical significance in addition to statistical significance. Proper design of PA interventions (45–90 min per session, more than 3 times per week and for 8–12 weeks) may enhance their effectiveness in treating SCI in children with ASD.

## 1 Introduction

The earliest child found to have autism was reported by American child psychiatrist Leo Kanner in 1943 [1], who described a child with self-centred behaviour and expression disconnected with society. Autism was first defined in 1967 in the Eighth Revision of the International Classification of Diseases (ICD-8), where it was listed as “infantile autism” as a subgrouping of schizophrenia. Later, in 1980, the Third Edition of the Diagnostic and Statistical Manual of Mental Disorders (DSM-III) [2] established autism as its own separate diagnosis, describing it as a “pervasive developmental disorder” distinct from schizophrenia. DSM-IV continued the designation “autistic disorder” as one of five subtypes of “pervasive developmental disorders,” while DSM-5 combined all of these subtypes into the umbrella term, “autism spectrum disorder (ASD).”

ASD is a neurodevelopmental disorder that typically manifests in early childhood, characterized by verbal and non-verbal social interaction deficits and restricted, repetitive patterns of behavior, interests, or activities. These clinical features can significantly impair daily functioning and might lead to lifelong neurodevelopmental challenges [3,4]. In accordance with the latest data from the Centres for Disease Control and Prevention, from 1975 to 2023, the incidence of children with ASD has increased from 0.002 % to 0.027 % [5]. Social Communication Impairment (SCI), a key clinical manifestation of ASD, has long been a central focus in the rehabilitation of children with ASD [6]. Commonly characterized by difficulties in social interaction, SCI primarily encompasses persistent challenges in utilizing language and non-verbal communication, impacting the quality of family life to different extents [7,8]. These findings highlight the significant global public health concern posed by the high incidence rate of ASD [9].

With continuous development of diagnosis and recognition techniques for ASD as well as research on the etiology of ASD in children, extensive intervention methods for ASD have emerged. These have included applied behavior analysis, prophylaxis, Denver Early Intervention Model, Interpersonal Development Intervention Plan, Floor Time, music therapy, drug treatment, and Sensorineural Auditory System [10]. Despite the limitations of current treatment methods for children with ASD, recent research has shown that physical activities (PA) interventions could have significant positive impact on the behavioural performance and overall development of children with ASD [[11], [12], [13], [14], [15], [16], [17]]. In comparison with other methods, PA would provide such children with a more functional therapeutic environment as well as diverse intervention forms [18]. There are currently multiple case and group studies on treating SCI in children with ASD by means of PA. A three-month case study conducted by Qian observed conspicuous progress in social interaction after adaptive sport activities among subjects with ASD [19]. Zhao et al. explored changes in social and communication behaviors during a 16-week horse-riding intervention, and found that horse-riding exercise significantly improved the social skills of children with ASD. Although different PA interventions have been found to be beneficial in improving the SCI of children with ASD, factors such as the dose of the intervention can produce different results, creating an element of uncertainty in the outcome of the intervention [20].

In summary, PA intervention may be an effective means to improve the SCI of children with ASD, but there are two limitations to its use: First, sports rehabilitation activities for children with ASD, such as swimming, equestrian training, roller skating, and other sports, entail high costs and a need to meet the unique needs of children with ASD, often with complex venue equipment, implementation difficulty, safety considerations, and other problems, making it difficult to practically execute and promote these interventions. Second, studies using other PA as interventions have not found a more comprehensive and prominent effect on the improvement of SCI in children with ASD. Therefore, we conducted a meta-analysis to quantify the impact of PA on SCI in children with ASD, to allow further exploration of the effectiveness of PA interventions. The existing research evidence has not fully clarified the neural mechanisms behind the improvement of SCI with PA interventions in ASD. By identifying more appropriate session duration, session frequency, and overall duration of exercises interventions, updated evidence on practical tools to improve SCI in children with ASD could be provided, ultimately enabling children with ASD to better integrate into society.

## 2 Materials & methods

### 2.1 Protocol

This systematic review adhered strictly to the Preferred Reporting Items for Systematic Reviews and Meta-analyses (PRISMA) guidelines [21], which enjoy widespread recognition as the established standard for reporting systematic reviews and meta-analyses. The protocol for this systematic review was registered in advance on the Open Science Framework (OSF) platform under the code number DOI 10.17605/OSF.IO/K6BFZ(accessed on July 19, 2023).

### 2.2 Literature screening

The study was conducted according to PRISMA guidelines and the literature was screened by two reviewers according to eligibility criteria. The literature was imported into Endnote X9 software for de-duplication, the titles and abstracts were read for initial screening, and the remaining literature was downloaded in full and screened. The extracted literature was then compared by two of the study authors (K.Q., Q.X.), the decision to include the article was made through a joint discussion with a third researcher.

### 2.3 Information source and searching strategy

The databases CNKI, Wanfang Data Knowledge Service Platform, Web of Science, PubMed, Cochrane Library were searched systematically from inception to September 11, 2024.

The Chinese search terms used for this study were ‘autism/autism’, ‘social/social communication disorder/social disorder/social skills/social ability’, and ‘sports intervention/exercise intervention/physical exercise/physical activity’, while the English language terms were ‘Autism Spectrum Disorder’ ‘Social communication disorder OR social disorder OR social skills’ ‘Physical exercise intervention OR exercise intervention OR exercise intervention OR physical exercise OR physical activity’. Taking PubMed as an example, the specific search formula is demonstrated in Table 2.

**Table 1:** Eligibility criteria.

	Inclusion Criteria	Exclusion Criteria
Population	The study encompassed children with ASD populations with no restrictions on sex or clinical conditions.Since children with ASD are a special group, Children who are diagnosed with autism are eligible for this article.	This meta specifically excluded non children with ASD. Children with ASD behave differently from normal children. Therefore, they were excluded from this study.
Intervention	The meta-study children with ASD who received PA intervention, The interventions consisted of exercise therapy, including aerobic exercises, anaerobic exercises, and structured exercises etc. without imposing any limitations on the duration or frequency of the training programs. Moreover, there were no constraints on the training volume or intensity, allowing for a comprehensive analysis of the effects of such training on the participants.	This meta does not include intervened by other non-PA interventions or routine activities. Additionally, participants who were exposed to a combination of PA and other training interventions were not considered within the scope of this meta.
Comparator	Control groups (not exposed to other training interventions, while retaining their regular activity levels and lifestyle) or active control groups (exposed to other exercise programs, not including exercise training)	The meta excluded participants who were already exposed to training programs incorporating PA. The primary objective was to assess the effects of structured PA training programs in comparison to other control conditions, making sure to exclude any potential overlap with participants who were already engaged in similar PA programs.
Outcomes	The assessment tools included scales related to the severity of social impairments in children and adolescents with ASD, such as the Social Responsiveness Scale, the Autism Diagnostic Observation Schedule, and the Autism Behavior Checklist. These scales represent ASD-specific behaviors, including social cognition, social awareness, social behavior, and social communication, with raw scores on the scales used as outcome measures.	The meta excluded studies literature results did not show the required indicators (e.g.Social cognition、Social motivation、Social awareness etc.)
Study design	Randomized controlled trials with no restriction on blinding methods.	The meta excluded studies that did not utilize randomized designs or controlled designs.

**Table 2:** Retrieval.

Retrieval process
#1 ((((("Autism Spectrum Disorder" [Mesh]) OR (Autism Spectrum Disorder [Title/Abstract])) OR (Autism Spectrum Disorders [Title/Abstract])) OR (Autistic Spectrum Disorder [Title/Abstract])) OR (Autistic Spectrum Disorders [Title/Abstract])) OR (Disorder, Autistic Spectrum [Title/Abstract]) #2 ("Child" [Mesh]) OR (children [Title/Abstract]) #3 (((((((((((((((((((((((((("Exercise" [Mesh]) OR (Exercise [Title/Abstract])) OR (Exercises [Title/Abstract])) OR (Physical Activity [Title/Abstract])) OR (Activities, Physical [Title/Abstract])) OR (Activity, Physical [Title/Abstract])) OR (PA [Title/Abstract])) OR (Exercise, Physical [Title/Abstract])) OR (Exercises, Physical [Title/Abstract])) OR (Physical Exercise [Title/Abstract])) OR (Physical Exercises [Title/Abstract])) OR (Acute Exercise [Title/Abstract])) OR (Acute Exercises [Title/Abstract])) OR (Exercise, Acute [Title/Abstract])) OR (Exercises, Acute [Title/Abstract])) OR (Exercise, Isometric [Title/Abstract])) OR (Exercises, Isometric [Title/Abstract])) OR (Isometric Exercises [Title/Abstract])) OR (Isometric Exercise [Title/Abstract])) OR (Exercise, Aerobic [Title/Abstract])) OR (Aerobic Exercise [Title/Abstract])) OR (Aerobic Exercises [Title/Abstract])) OR (Exercises, Aerobic [Title/Abstract])) OR (Exercise Training [Title/Abstract])) OR (Exercise Trainings [Title/Abstract])) OR (Training, Exercise [Title/Abstract])) OR (Trainings, Exercise [Title/Abstract]) #4 #1 and #2 and #3

### 2.4 Qualification criteria

The PICOS framework was utilized to ensure that a standardized approach was used to formulate our clinical research question, thoroughly search the literature to answer that question, and generate an evidence-based answer to the query in a robust and reproducible manner (Table 1).

### 2.5 Data extraction and coding

In accordance with the inclusion criteria, the data extraction information is shown in Table 3.

**Table 3:** Data extraction information details.

Included entries	Include specific information
essential information	(1) Name of the author; (2) Year of publication; (3) Age of subjects
other information	(1) Sample size of experimental group and control group (2) The outcome indicators of the experimental group and the control group, the relevant mean and standard deviation (3) Because the included literature was RCT, only the intervention measures of the experimental group were included, including the cycle, frequency and cycle of the intervention (4) Test tools for outcome indicators

After extracting relevant data, 2 researchers screened the titles and abstracts of all retrieved articles independently. In the event of discrepancies, discussions would be held to reach a consensus on which articles required full-text screening. A third researcher would be consulted to make the final decision if necessary. Subsequently, articles were fully screened for incorporation by 2 researchers independently. The research team maintained a collaborative approach to resolve any differences in data interpretation. If there were any missing data in the reviewed literature, the corresponding author would be contacted via email to obtain necessary information. Where data were only presented in graphical form, the Web Plot Digitizer software was utilized to extract the mean and standard deviation values.

### 2.6 Quality evaluation of literature

The quality of the included literature was assessed using the Cochrane bias risk evaluation criteria, which includes factors such as random allocation methods, allocation scheme concealment, blinding method for subjects and experimenters, blinding method for outcome evaluation, integrity of result data, and selective reporting and other sources of bias.

### 2.7 Statistical analysis

Among the nine studies reviewed in this study, there were differences in the measurement dimensions of individual studies and those measurement dimensions required merging in the process of extraction. As the data are presented as continuous variables, they need to be converted according to the public announcement. Set the sample size of measurement method A as N_1_, mean as M_1_, and standard deviation as SD_1_; the sample size of measurement mode B be N_2_, mean M_2_, and standard deviation SD_2_, then the combined sample size N=N_1_+N_2_, mean M=(N_1_M_1_+N_2_M_2_)/(N_1_+N_2_), and:

$$SD=\sqrt{\frac{\left(N_{1}-1\right){SD}_{1}^{2}+\left(N_{2}-1\right){SD}_{2}^{2}+\frac{N_{1}N_{2}}{N_{1}+N_{2}}\left(M_{1}^{2}+M_{2}^{2}-2M_{1}M_{2}\right)}{N_{1}+N_{2}-1}}$$

If there were multiple dimensions of data to be combined, the data of the first two dimensions would be consolidated first according to the above formula, then the obtained data integrated with the third dimension, and so forth. In the range of 75%–100 %, possibilities of extremely high heterogeneity would exist [22]. The combined effect values Z, weighted mean difference (WMD) and confidence interval values (95 % CI) of the reviewed literature were analysed by selecting an area of heterogeneity based on the results of the heterogeneity test for the effects model. If heterogeneity existed, a subgroup or sensitivity analysis of the source of heterogeneity would be applied, and the heterogeneity sources would be treated analytically to reduce heterogeneity. Forest plots were used to test the reviewed literature for publication bias.

## 3 Results

### 3.1 Study identification and selection

**Fig. 1:**
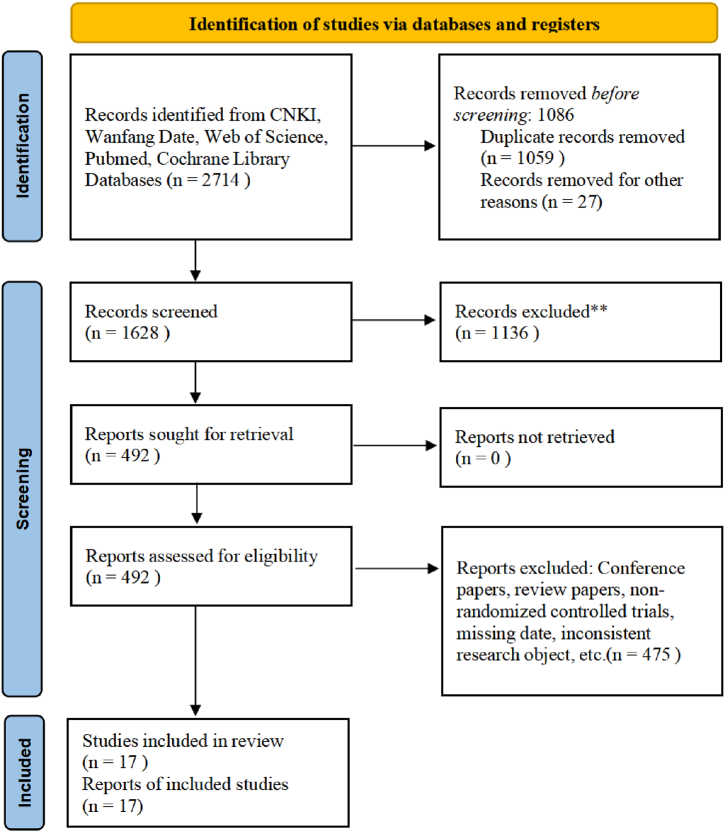
Flow diagram of literature search.

The initial search yielded a total of 2714 titles, as illustrated in Fig. 1.

### 3.2 Characteristics of included literature

Altogether 710 participants from 17 studies published by 2024 were included in this study by systematic search and other sources, all of which were conducted on children diagnosed with ASD (Table 4).

**Table 4:** Included basic information.

Literature	Age (year)	Sample size (T/C)	Experimental group score	Control group score	Intervention	Frequency (times/week)	Time/min	Cycle /week	Assessment tool	Diagnosis of ASD
Yang et al. (2016)	3–10	(40/40)	15.12 ± 5.84	19.24 ± 2.35	exercise intervention	6	90	24	ATEC scale	ICD-10
Ren (2018)	3–14	(35/35)	82.26 ± 10.6	75.98 ± 11.2	somatosensory game intervention	3	30	8	PEDI scale CARS scale	DSM-IV
Liu et al. (2021)	6–10	(13/10)	82.92 ± 15.86	92.80 ± 0.99	Big muscle games	4	60	6	SRS-2; ASSS	NA
Janice et al. (2021)	8–11	(14/20)	76.36 ± 14.48	72.25 ± 18.19	Comprehensive Combat Intervention	2	45	13	SSIS	NA
Wang et al. (2020)	3–6	(18/15)	82.22 ± 27.55	95.93 ± 19.47	Mini-basketball sports intervention	5	40	12	SRS-2	DSM-Ⅴ
Cai et al. (2020)	3–6	(15/14)	15.44 ± 8.33	18.19 ± 8.78	Mini-basketball sports intervention	5	40	12	SRS-2	DSM-Ⅴ
Yang et al. (2021)	3–6	(15/15)	13.24 ± 6.26	15.05 ± 7.27	Mini-basketball sports intervention	5	40	12	SRS-2	DSM-Ⅴ
DITZA et al. (2022)	3–7	(30/21)	63.2 ± 11.13	67.89 ± 13.61	Outdoor adventure exercise intervention	1	30	13	SRS; VABS-II	DSM-Ⅴ
Xu et al. (2019)	3–14	(50/53)	33.14 ± 5.76	35.59 ± 5.51	Sensory Integration Training (SIT)	3	30	60	CARS	CCMD-3
Yang et al. (2024)	3–6	(15/15)	82.5 ± 29.55	97.3 ± 21.35	Mini-basketball sports intervention	5	40	12	SRS-2	DSM-Ⅴ
Amir et al. (2022)	6–10	(8/8)	16.37 ± 14.62	15.37 ± 12.65	Combined Physical Training Strategies	3	60–80	8	GARS-2 scale	NA
Qi et al. (2024)	3–6	(27/14)	83.21 ± 22.95	96.36 ± 22.86	Recreational ball games	5	40–45	12	SRS-2	DSM-Ⅴ
Cai et al. (2020)	3–6	(30/29)	82.80 ± 27.83	95.47 ± 22.30	Mini-basketball sports intervention	5	40	12	SRS-2	DSM-Ⅴ
Mahboubeh et al. (2018)	5–12	(12/14)	14.58 ± 7.79	21.28 ± 8.87	SPARK	3	40	12	GARS-2	DSM-IV
Mirella and Silvano (2019)	3–8	(13/12)	91.31 ± 24.31	97.75 ± 27.50	Water Sports	0.5	30	24	SRS	DSM-Ⅴ、DSM-IV
Giovanni et al. (2018)	6–13	(13/13)	66.15 ± 11.39	61 ± 7.25	Water Sports	2	45	40	VABS	DSM-V
Margaret et al. (2009)	6–12	(19/15)	73.6 ± 24.1	94.4 ± 32.1	Equestrianism	1	60	12	SRS-2	DSM-IV

ATEC:Autism Treatment Assessment Scale; ICD-10:International classification of diseases 10 Version; PEDI:Rating Scale for Reduced Ability Children; CARS:Autism Symptom Rating Scale for Children; DSM-IⅤ:Diagnostic and Statistical Manual of Mental Disorders, 4th Edition; DSM-Ⅴ:Diagnostic and Statistical Manual of Mental Disorders, 5th Edition; SSIS:Social Skills Improvement System; SRS-2:Social Responsiveness Scale Second EditionSRS:Social Responsiveness Scale; ASSS:Social skills rating scale for children with autism; VABS:Vineland Adaptive Behavior Scales.

### 3.3 Quality evaluation

**Fig. 2:**
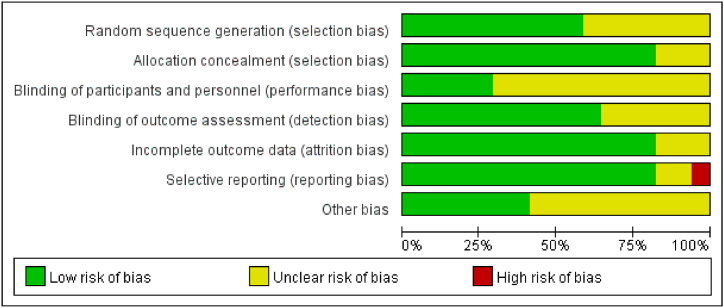
The quality evaluation is shown.

The quality evaluation is shown in Fig. 2. Cochrane Risk of Bias Assessment Tool were adopted for quality assessment of methodological quality. The quality scores of the included studies were mostly assigned to grade A\B, all of which were at the medium and high quality level, as shown in Fig. 2. The overall methodological quality of the included literature quality was good. Finding explicitly stated information in articles regarding random sequence generation (i.e., specifically how randomization was achieved) and allocation concealment is rare.

### 3.4 Overall effect of exercise on social disorder in children with ASD

**Fig. 3:**
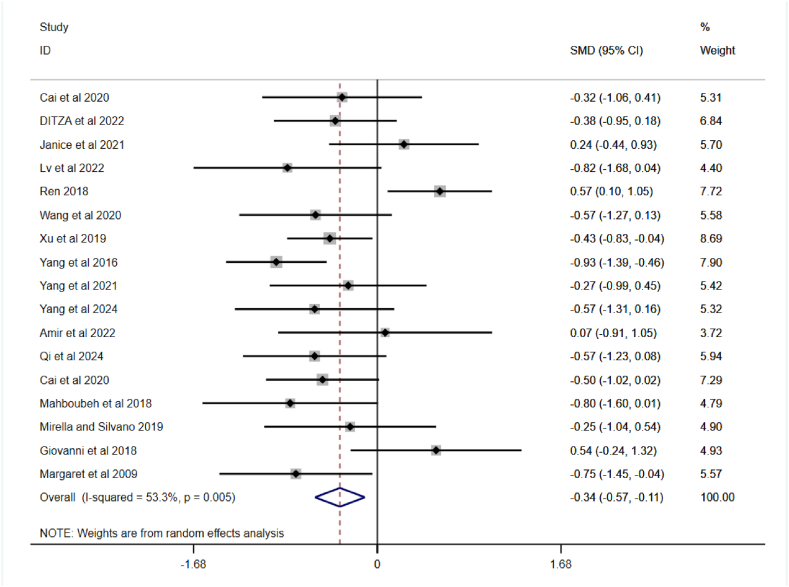
A meta-analysis of the overall effects of PA on SCI in children with ASD.

A total of nine randomised controlled studies systematic review and meta-analysis to elaborate and compare the effects of different PA on SCI in children with ASD. Fig. 3 shows the results of the meta-analysis of sports interventions for SCI in children with ASD, which revealed a heterogeneity test of I^2^ = 53.3 %, p < 0.01, indicating a medium degree of statistical heterogeneity between studies. Random effects modeling was therefore used to analyse the data. The meta-analysis results demonstrated a combined effect size of SMD = −0.34, 95 % CI: −0.57, 0.11, P < 0.01, which indicates a statistically significant difference, i.e. indicating that PA could effectively improved of SCI in children with ASD compared to the control group.

It is noteworthy that in Fig. 3, the “experimental” refers to the experimental group included in the literature, which engaged in different PA compared to the control. Participants in the control group, on the other hand, engaged only in regular activities. Additionally, the included values represent the post-test scores on different SCI scales for the experimental and control groups, providing a clear reflection of the intervention effects of PA.

### 3.5 Subgroup moderation effect test

**Fig. 4:**
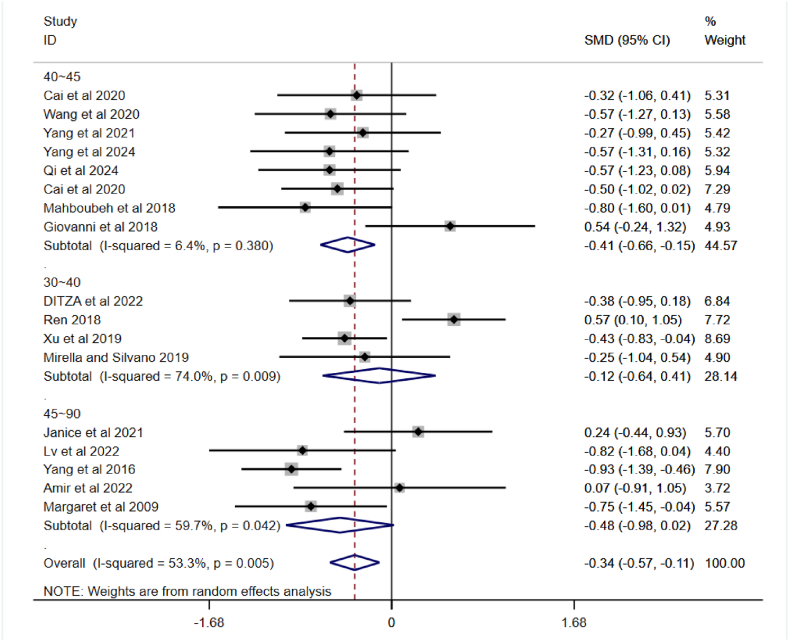
A subgroup analysis of intervention duration on SCI in children with ASD.

**Fig. 5:**
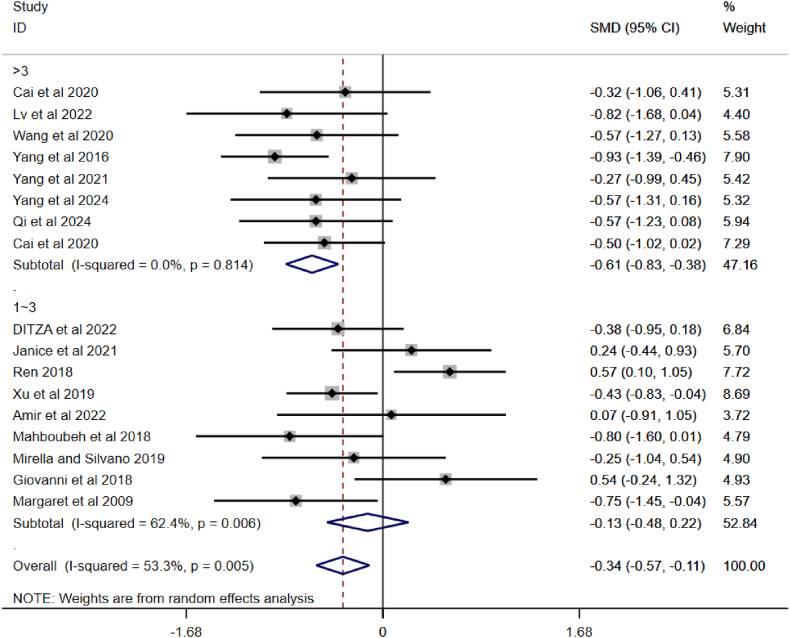
A subgroup analysis of intervention frequency on SCI in children with ASD.

**Fig. 6:**
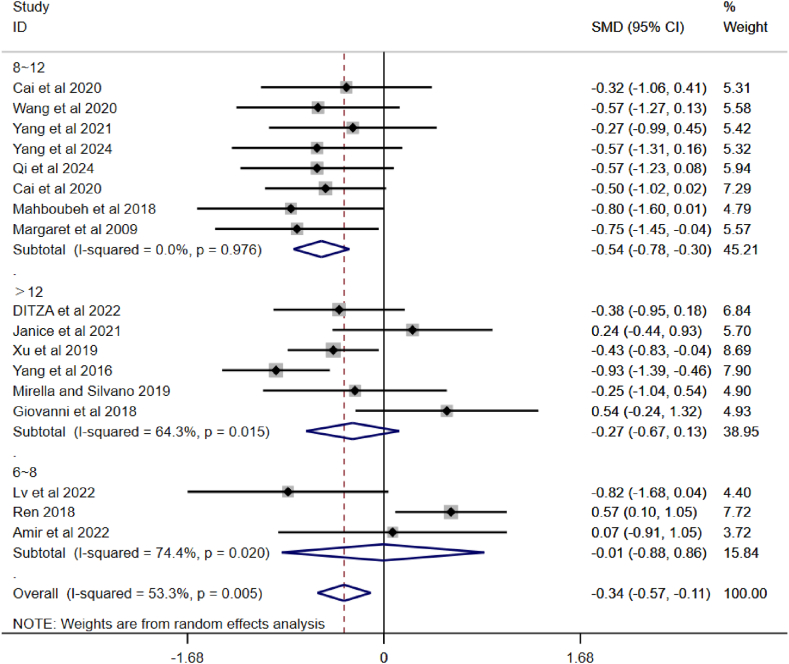
A subgroup analysis of intervention cycle on SCI in children with ASD.

Studies have found that PA can effectively improve the SCI of children with ASD. As the forest plot showed a high level of heterogeneity, a subgroup analysis was applied to the incorporated articles and the outcomes are shown in Fig. 4, Fig. 5, Fig. 6. The observations are as follows:

Fig. 4 shows, the overall pooled effect is SMD = −0.34, 95 % CI (−0.57, −0.11), I^2^ = 53.3 %, p = 0.005, indicating some heterogeneity, but overall PA interventions have a positive effect on SCI in children with ASD (since SMD is negative and the confidence interval does not include 0). The 30–40min group exhibited high heterogeneity, the 45∼90min group also showed moderate heterogeneity, while the 40∼45min group had almost no heterogeneity. Overall, PA interventions have a positive effect on SCI in children with ASD, with the most significant effect observed in interventions lasting 40–45 min.

Fig. 5 shows that the overall pooled effect is SMD = −0.34, 95 % CI (−0.57, −0.11), I^2^ = 53.3 %, p = 0.005, indicating some heterogeneity, but overall PA interventions have a positive effect on SCI in children with ASD (since SMD is negative and the confidence interval does not include 0). The 1–3 intervention frequency group exhibited high heterogeneity, while the group with >3 interventions had almost no heterogeneity. PA interventions with a frequency of >3 times a week significantly improved SCI in children with ASD (SMD = −0.61). The effect of 1∼3 interventions was not significant (SMD = −0.13).

Fig. 6 shows that the overall pooled effect is SMD = −0.34, 95 % CI (−0.57, −0.11), I^2^ = 53.3 %, p = 0.005, indicating some heterogeneity, but overall PA interventions have a positive effect on SCI in children with ASD (since SMD is negative and the confidence interval does not include 0). The 6–8 week group exhibited high heterogeneity, the >12-week group exhibited moderate heterogeneity. The 8–12 week group had almost no heterogeneity. Therefore, PA interventions lasting 8–12 weeks have a significant positive effect on SCI in children with ASD (SMD = −0.54).

In general, the optimal dose of PA interventions consisted of an intervention period of 8–12 weeks, over 3 interventions per week, and 45–90 min per session. As the results of the subgroup analysis were not able to demonstrate the effect of different PA or the effect of exercise dose on the results of the experiment, a more in-depth discussion remains necessary on how PA interventions can improve SCI in children with ASD under different circumstances.

### 3.6 Sensitivity analysis

**Fig. 7:**
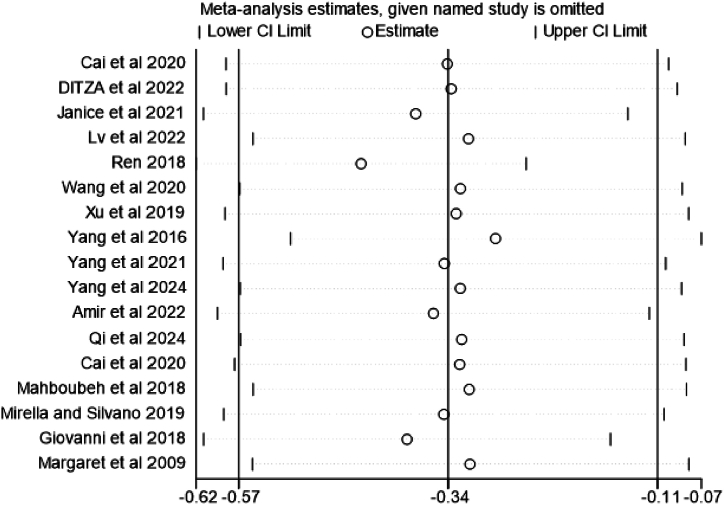
Sensitivity analysis.

To investigate whether heterogeneity across studies was caused by specific studies, a sensitivity analysis was performed on highly heterogeneous PA interventions for SCI in children with ASD. Fig. 7 shows the sensitivity analysis of this study, aimed at assessing the impact of each individual study on the overall Meta-analysis results. The center line (vertical line) represents the overall Meta-analysis effect estimate (about −0.34) when all studies are included. If a circle and its 95 % confidence interval line significantly differ from the center line's overall effect estimate, it indicates that the study has a substantial impact on the overall Meta-analysis results. From the figure, it can be seen that after removing any single study, the estimated effect size does not change significantly, and all estimates are roughly concentrated around the vertical line (−0.34). This indicates that the results of the meta-analysis are relatively robust, and individual studies have little impact on the overall results. The sensitivity analysis shows that the conclusions of the meta-analysis will not undergo major changes due to the removal of a single study.

### 3.7 Publication bias test

**Fig. 8:**
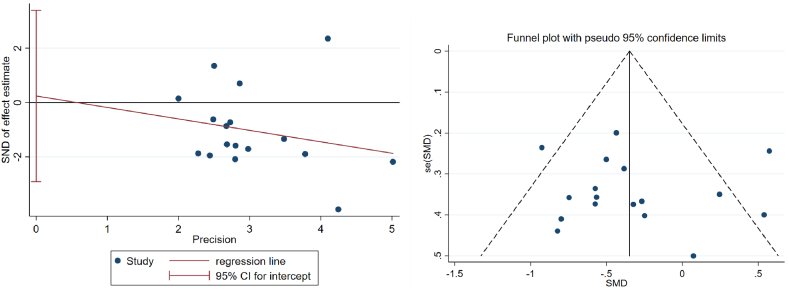
Publication bias funnel plots of included studies and results of Egger's linear regression method.

To evaluate publication bias in the Meta-analysis, we created a funnel plot (as shown in Fig. 8). Ideally, if there is no publication bias, the data points should symmetrically distribute around the center line and fall within the pseudo 95 % confidence intervals. The figure shows that although the data points are somewhat dispersed, there is no significant skewness overall, indicating no significant publication bias. Most data points are concentrated at the top of the funnel plot, indicating that these studies have high precision. Additionally, some data points are located outside the funnel plot, indicating some heterogeneity. Thus, the Egger's linear regression test was continued in a quantitative manner (Fig. 8), the P-value for bias is 0.873, which is greater than 0.05, indicating that there is no publication bias in this article. Therefore, despite some heterogeneity, our Meta-analysis results are generally robust.

## 4 Discussion

With eleven studies from recent years (by 2024) included in this meta-analysis and an overall favorable quality assessment, our research is timely and innovative in examining the role of PA in improving SCI in children with ASD. Compared to other treatments, there is a paucity of international research on PA interventions for SCI, especially among children with ASD, as well as an absence of evidence-based studies that specifically address SCI in ASD.

The application of PA interventions in the rehabilitation of ASD has received considerable research attention, with increasingly promising results [[23], [24], [25], [26], [27], [28], [29]]. Our meta-analysis of PA interventions for children with ASD has shown an overall positive effect of PA on SCI that is correlated with the dose of PA intervention. The experimental interventions included in the literature in this study included mini-basketball, sensory training, large muscle exercises and mixed martial arts etc, demonstrating a diversity of PA. By modifying sport rules to accommodate the developmental characteristics of children with ASD, it is possible to bring back the game nature of sport as a way of helping children with ASD to recover from SCI. According to Wang, SCI in children with ASD could affect family quality of life and have a negative impact on their future employment and careers [30]. Since they may not be able to concentrate on PA for long periods of time, it is important to choose the right dosage for PA interventions. This study revealed that the optimal dose of PA intervention for improving SCI in children with ASD was between 40 and 45 min per session and that the session period should be maintained for 8–12 weeks.

In terms of the intervention duration, it was argued that as children with ASD are relatively non-compliant and less execute with PA interventions than healthy children, the same intervention duration could have variable effects, with 40–45 min of PA intervention at each session showing the greatest effect. Furthermore, previous studies have found that a longer duration of intervention may also have a positive impact on SCI in children with ASD. For example, one study, guided by adaptive sports and early intervention theory for children with ASD, showed that an appropriate dose (1 h three times per week for 18 weeks) of a basketball exercise intervention led to significant improvements in SCI in 8 school-aged young students with ASD [31]. These improvements are thought to be associated with increased opportunities for social interaction and the development of motor skills, which are critical for facilitating communication and social engagement in these children.

In terms of the intervention frequency, the literature included in this study mostly used interventions with a frequency of >3 times per week, which were more effective in improving SCI in children with ASD and are in line with the findings of previous studies. Using a sports game intervention five times per week for three months, Zhang and his team were able to demonstrate a remarkable increase in active communication in children with ASD [32]. These same researchers showed that an approach utilizing over 3 PA interventions per week significantly attenuated a child's symptomatic problem behaviour after 3 months [33]. Consistently, that high-frequency interventions not only enhance social interaction but also promote overall well-being and physical fitness in children with ASD. This evidence suggests that frequent, structured physical activities may provide a predictable routine and increased opportunities for practice, which are crucial for reinforcing positive behavioral changes and skills development in this population.

In terms of intervention cycle, the reviewed studies showed that the total duration of the PA intervention (in weeks) was positively associated with the intervention effect within a certain range. This is consistent with previous research demonstrating a significant impact of appropriate intervention length on improvements in SCI in children with ASD. For example, one study of a 10-week intervention using THR for pre-schoolers and juveniles with ASD discovered that individuals with ASD who completed 10 weeks of THR exhibited significant improvements in irritability, lethargy, stereotyped behaviour, hyperactivity, expressive language skills, motor skills and language practice/motor planning skills [34]. Najafabadi et al. conducted a sports intervention (SPARK training) on children with ASD over 36 sessions, and found that this training was effective not only for motor enhancement but also for improving social skills [35]. Gabriels utilized 12 weeks of horse-riding training in an effort to improve the social functioning of 34 children (aged 4–10 years) with ASD, and found that subjects in the THR group exhibited greater social motivation and less inattention, distraction and sedentary behaviour [36]. Specifically, interventions lasting between 8 and 12 weeks were consistently found to be the most effective duration, providing ample time for participants to adapt to the activities and for sustained behavioral changes to take place. The effectiveness of this timeframe may be attributed to the balance it offers between sufficient exposure to the intervention and maintaining participant engagement and motivation, avoiding the fatigue or burnout that may occur with longer programs.

Various types of PA have been studied to address social and other disorders in children with ASD. For example, in addition to those referenced earlier, other horse-riding intervention studies have also demonstrated beneficial effects on children with ASD [37,38], suggesting that THR could be an effective means to improve social functioning in children with ASD. In a 14-week karate exercise program, Bahrami et al. investigated the effects of karate on children with ASD and found a significant reduction in communication deficits in the exercise group compared to the control group, with sustained improvement in follow-up interviews 1 month after intervention [39]. Zhu et al. recruited 36 children with ASD aged 6–12 years and randomised them to a 20-week mini-basketball exercise intervention versus a control intervention. The mini-basketball exercise intervention enhanced the working mnemonic performance of children with ASD [40]. Based on this, it is possible that different approaches could be aligned with PA interventions and have a more positive impact on the SCI of children with ASD. The results of this study may be biased to some extent and subsequent studies should include more relevant literature to verify this finding.

The Discussion cites a number of studies that ostensibly support the authors’ findings that PA interventions may improve SCI in children with ASD. While these citations do help place the research findings into some helpful context, there are certain limitations that need to be addressed. These include the high level of statistical heterogeneity observed, the relatively small sample sizes of the individual studies included in the meta-analysis, and the possible lack of standardization in terms of how particular PA interventions were implemented. Additionally, the overall effect size, while statistically significant, was relatively modest (−0.34), suggesting that the practical significance of these findings may be limited.

Future studies should aim to explore the mechanisms underlying the observed improvements, which could provide deeper insights into how these interventions benefit SCI in children with ASD. Investigating the optimal timing for interventions could also be crucial in maximizing their effectiveness, determining whether early, middle, or late-stage interventions yield the best outcomes. Furthermore, comparing the impacts of different types of PA on SCI could identify which activities are most beneficial, thereby informing tailored intervention strategies.

## 5 Conclusions

As a core symptom of children with ASD, SCI significantly affect their well-being, underscoring its critical importance. Although PA interventions cannot completely cure children with ASD, they could play a profound role in helping them to develop healthy personalities, improve their social functioning and alleviate the negative effects during their growth and development. This meta-analysis revealed a modest effect size for PA interventions in improving social communication impairments in children with ASD. Acknowledging the study's limitations is crucial, including moderate statistical heterogeneity and small sample sizes. Therefore, further research is needed to address these limitations and to more definitively determine whether PA interventions can significantly improve SCI in children with ASD.

## CRediT authorship contribution statement

**Kai Qi:** Writing – original draft, Visualization, Validation, Software, Resources, Methodology, Investigation, Funding acquisition, Formal analysis, Data curation, Conceptualization. **Xiaoshuang Wang:** Writing – review & editing, Supervision. **Qi Xu:** Writing – original draft, Software, Data curation. **Bingyu Hu:** Writing – original draft. **Ziyi Wang:** Writing – original draft. **Marcin Białas:** Writing – review & editing, Supervision, Methodology.

## Consent for publication

Not applicable.

## Data availability statement

The data supporting the findings of this study are available within the article. Any additional data or materials can be requested from the corresponding author.

## Fund project

There is no funding to report.

## Declaration of competing interest

The authors declare that they have no known competing financial interests or personal relationships that could have appeared to influence the work reported in this paper.
